# Effects of acupuncture at Pericardium-6 and Stomach-36 on nausea, sedation and gastrointestinal motility in healthy dogs administered intravenous lidocaine infusions

**DOI:** 10.1371/journal.pone.0226065

**Published:** 2019-12-05

**Authors:** Mariko L. St. James, DeAnna L. Kosanovich, Lindsey B. Snyder, Qianqian Zhao, Brian G. Jones, Rebecca A. Johnson

**Affiliations:** 1 Department of Surgical Sciences, University of Wisconsin, Madison, Wisconsin, United States of America; 2 Department of Biostatistics and Medical Informatics, University of Wisconsin, Madison, Wisconsin, United States of America; 3 Antech Imaging Services, Fountain Valley, California, United States of America; University of Texas Medical Branch, UNITED STATES

## Abstract

The objectives of this study were to assess gastrointestinal transit times, sedation, and signs of nausea associated with intravenous lidocaine infusions in dogs following targeted acupuncture at Pericardium-6 (PC6) and Stomach-36 (ST36). In a randomized, blind crossover design, 6 healthy, adult Beagles were fed thirty 1.5 mm barium-impregnated polyethylene spheres (BIPS), then were subject to 30 minutes of: 1) no acupuncture, 2) bilateral targeted acupuncture at PC6 and ST36, or 3) bilateral non-target acupuncture at Lung-5 (LU5) and Bladder-55 (BL55). Lidocaine was immediately administered at 1 mg/kg intravenously followed by 50 μg/kg/min. BIPS were tracked radiographically; sedation and nausea were scored at baseline (Time 0) and for 11 hours during lidocaine infusions. Transit times and sedation and nausea scores were analyzed with a linear mixed-effects model; the number of BIPS at defined time points was analyzed with a piecewise linear mixed-effects model. All P values were two-sided and P < 0.05 was considered significant. Sedation and nausea scores did not differ between treatments at any time point (all P > 0.05). However, nausea scores in all groups were significantly greater at Times 5 through 7 and at Time 11 compared to Time 0 whereas sedation scores in all groups were significantly greater at Times 2 through 11 compared to Time 0 (all P < 0.05). The number of BIPs found out of the stomach, the number found in the large intestine, gastric emptying and gastrointestinal transit times did not differ between treatments (all P > 0.05). Acupuncture at PC6 and ST36 did not alleviate nausea and sedation associated with lidocaine infusions in clinically normal animals or affect gastric emptying and gastrointestinal transit.

## Introduction

Lidocaine is an amide local anesthetic and Class 1b antiarrhythmic agent used intravenously for its anti-inflammatory, antinociceptive, prokinetic, and antioxidant properties in horses and humans [1–5]. In addition, lidocaine infusions are frequently used in dogs to reduce nociceptive stimulation and the minimum alveolar concentration of inhalants peri-operatively [6–11]. In systemically healthy, conscious dogs, prolonged lidocaine constant rate infusions also transiently accelerate gastrointestinal (GI) transit; however, they cause significant sedation, nausea, and vomiting [12, 13], potentially limiting their usefulness in select patients.

Acupuncture is a minimally invasive treatment modality that uses acupoints specific to the area of interest. Acupoints, well described in Eastern medicine and increasingly accepted in Western medicine, are defined as neurovascular bundles surrounded by a connective tissue sheath with high density of A-delta and C fibers, arterioles, lymphatic vessels and mast cells. These points lie along meridians or divisions, which are thought to transmit energy to particular regions and correlate more closely with the anatomical structure of fascial planes than nerve pathways [14–18]. Acupuncture specifically directed at visceral acupoints such as Pericarium-6 (PC6) and Stomach-36 (ST36), is used to treat multifactorial nausea and GI dysmotility in both human and veterinary medicine [15–17, 19–24]. In addition, acupuncture at PC6 and ST36 transiently slowed GI transit in conscious, healthy dogs [25] and reduced nausea and vomiting associated with opioid (hydromorphone) administration [26]. However, other hypothesis-driven investigations that address the use of acupuncture to reduce nausea and vomiting associated with other clinically used pharmacologic agents are absent.

The study objective was to explore the efficacy of simultaneously administered acupuncture in dogs experiencing untoward effects associated with lidocaine infusions as a potential clinical tool. We hypothesized that targeted acupuncture at PC6 and ST36 (compared to non-target/SHAM or no acupuncture) would mitigate the GI effects associated with lidocaine constant rate infusions in conscious dogs such as nausea, vomiting, and alterations in GI transit time, but sedation would still be present in all groups. However, contrary to this hypothesis, targeted acupuncture did not reduce nausea and vomiting associated with lidocaine infusions in dogs, and gastric emptying and gastrointestinal transit were similar across targeted acupuncture, non-target (SHAM) acupuncture and no acupuncture (control) groups.

## Materials and methods

### Animals

Similar to previously published studies [13, 25], six young adult beagles (3 males, 3 females; 7–12 months old) with mean body weight 8.4±1.6 kg (mean±SD) were studied in a blind, crossover design. They were deemed healthy by physical examination, no clinical signs of GI disease (inappetence, vomiting, diarrhea), and normal packed cell volume, total protein, and blood urea nitrogen (Azostix; Siemens Healthcare Diagnostics Inc., Tarrytown, NY) values. They had not received previous acupuncture. This study was carried out with the recommendations in the Guide for the Care and Use of Laboratory Animals of the National Institutes of Health. The Institutional Animal Care and Use Committee in the School of Veterinary Medicine at the University of Wisconsin approved the protocol (Protocol Number: V5077). All efforts were made to ameliorate animal suffering.

### Experimental procedures

#### Feeding and baseline parameters

Dogs were allowed to acclimate to their housing environment and new feeding schedule for 7 days prior to study start. They were touched and their limbs manipulated twice daily to acclimate them to the acupuncture techniques. Similar to previously described techniques, dogs were held off feed 12 hours prior to the study then fed half of their resting energy requirements (0.5 X 70 X [body weight in kg]^0.75^) in kibble with 30 barium-impregnated polyethylene spheres (BIPS; Med I.D. Systems Inc., Grand Rapids, MI) in one teaspoon of baby food dispersed throughout [13, 25, 27, 28]. Immediately following feeding, they were scored for nausea and sedation; one investigator performed nausea scoring and a second performed the sedation scoring independently at baseline and throughout the experiment to reduce inter-scorer variability. Baseline (Time 0) three-view abdominal radiographs (ventrodorsal, right and left lateral) were then taken within 10 minutes after feeding to quantify ingested BIPS.

The nausea scale was adapted from previous publications to provide a thorough and detailed assessment with scores ranging from 0 to 11 [Table 1; 13, 29, 30]. As part of a separate study, two sedation scorings systems were used; the first scale was adapted from our previous investigation [13]. The second scale was a validated sedation scale in dogs with excellent internal consistency and very good reliability; scores ranged from 0 to 21 [Table 2; 31]. Both sedation scoring systems produced similar statistical results, and only the data from the validated system are reported here. Based on the sedation scoring system, a score of 0 to 3 was graded as little to no sedation, moderate sedation was assigned to scores between 4 and 11, and heavy sedation was present with scores greater than 13 [31].

**Table 1:** Nausea scoring assessed over 60 minutes in healthy dogs given targeted acupuncture (PC6, ST36), non-targeted acupuncture (LU5, BL55), or no acupuncture and a loading dose of 1mg/kg IV lidocaine followed by 50 μg/kg/min constant rate infusions.

Observation	Score	Criteria
**Behavioral**	0	No signs of nausea
	1	Licking or smacking lips, increased salivation, yawning,
		Murmuring
	2	Any of the above with exaggerated swallowing, belching, shortness of breath
	3	Any of the above with circling, restlessness, retching or vomiting
**Severity**	0	No apparent signs
	1	Mild signs, dog intermittently settles itself
	2	Moderate signs, dog can be comforted by verbal or tactile human interaction
	3	Severe signs, dog cannot be comforted by verbal or tactile human interaction
**Frequency of signs**	0	No signs
	1	Seldom/intermittent signs of nausea (detailed above)
	2	Continuous signs of nausea but no vomiting/retching
	3	One event of vomiting/retching
	4	Two events of vomiting/retching
	5	Three or more events of vomiting/retching

**Table 2:** Sedation scoring assessed over 5 minutes in healthy dogs given targeted acupuncture (PC6, ST36), non-targeted acupuncture (LU5, BL55), or no acupuncture and a loading dose of 1mg/kg IV lidocaine followed by 50 μg/kg/min constant rate infusions.

Observation	Score	Criteria
**Spontaneous posture**	0	Standing
	1	Tired but standing
	2	Lying but able to rise
	3	Lying but difficulty standing
	4	Unable to rise
**Palpebral reflex**	0	Brisk
	1	Slow but with full corneal sweep
	2	Slow but with partial corneal sweep
	3	Absent
**Eye position**	0	Central
	1	Rotated forward/downward but not obscured by third eyelid
	2	Rotated forward/downward and obscured by third eyelid
**Jaw/tongue relaxation**	0	Normal jaw tone/strong gag reflex
	1	Reduced tone/moderate gag reflex
	2	Much reduced tone/moderate gag reflex
	3	Loss of jaw tone/no gag reflex
**Response to noise (clap)**	0	Normal startle reaction (head turn toward noise/cringe)
	1	Reduced startle reaction (reduced head turn/minimal cringe)
	2	Minimal startle reaction
	3	Absent reaction
**Resistance when laid into**	0	Much struggling/not allowing to be put into position
**sternal recumbency**	1	Some struggling but allowing this position
	2	Minimal struggling/permissive
	3	No struggling
**General appearance and**	0	Excitable
**attitude**	1	Awake and normal
	2	Tranquil
	3	Stuporous

#### Acupuncture and lidocaine treatments

**Fig 1:**
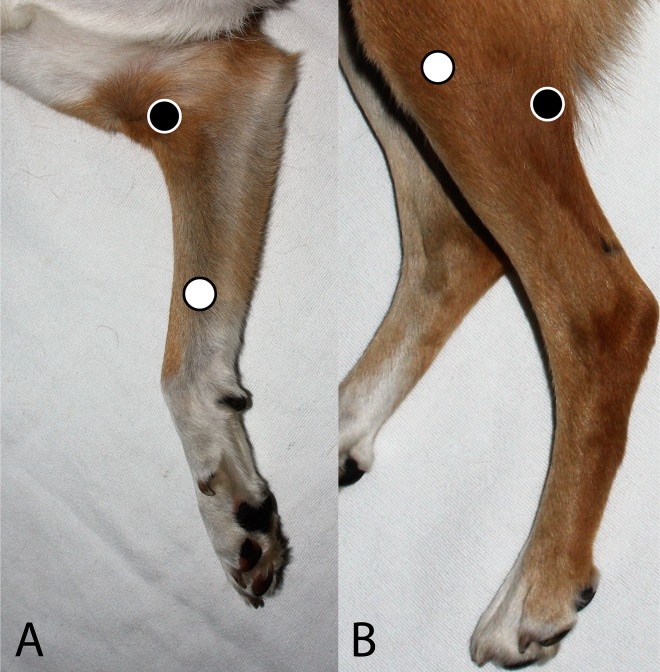
TARGET and SHAM acupoints. (A) TARGET acupoint PC6 (white circle, proximal to the carpus and between the flexor tendons), on the medial aspect of the canine thoracic limb and SHAM acupoint LU5 (black circle, lateral to the biceps brachii tendon) (B) TARGET acupoint ST36 (white circle, lateral to the tibial crest within in the cranial tibialis muscle), on the lateral aspect of the canine pelvic limb and SHAM acupoint BL55 (black circle, between the biceps femoris and semitendinosus muscles).

**Fig 2:**
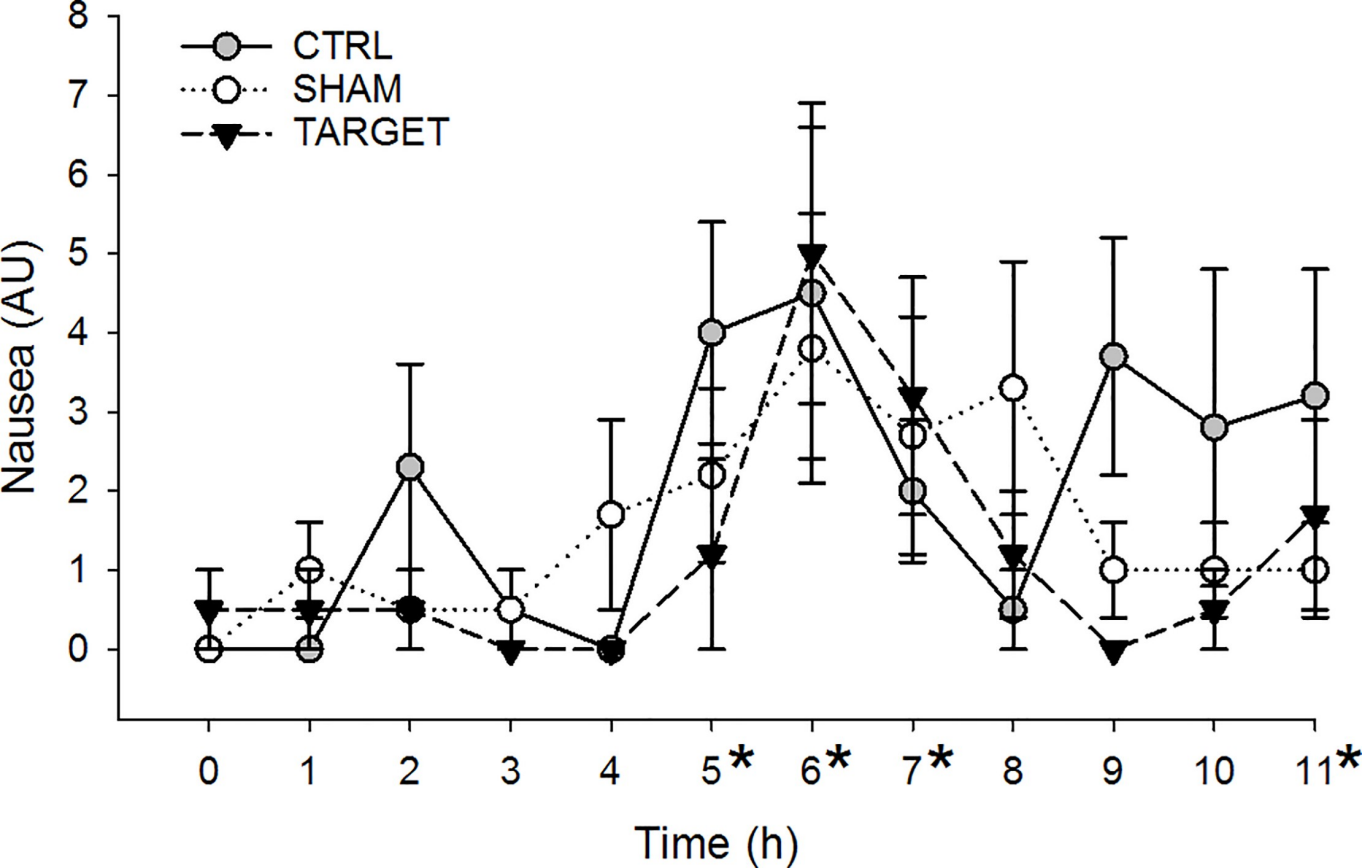
Nausea scores in healthy dogs given a loading dose of 1 mg/kg IV lidocaine followed by an infusion of 50 μg/kg/min. Dogs were either given no acupuncture (gray circles; CTRL), acupuncture at LU5 and BL55 (white circles; SHAM), or acupuncture at PC6 and ST36 (black triangles; TARGET). Scores ranged from 0 to 11 and are presented as mean ± SEM. No differences between treatment groups were present. *Significantly increased time point across treatment groups compared with Time 0.

**Fig 3:**
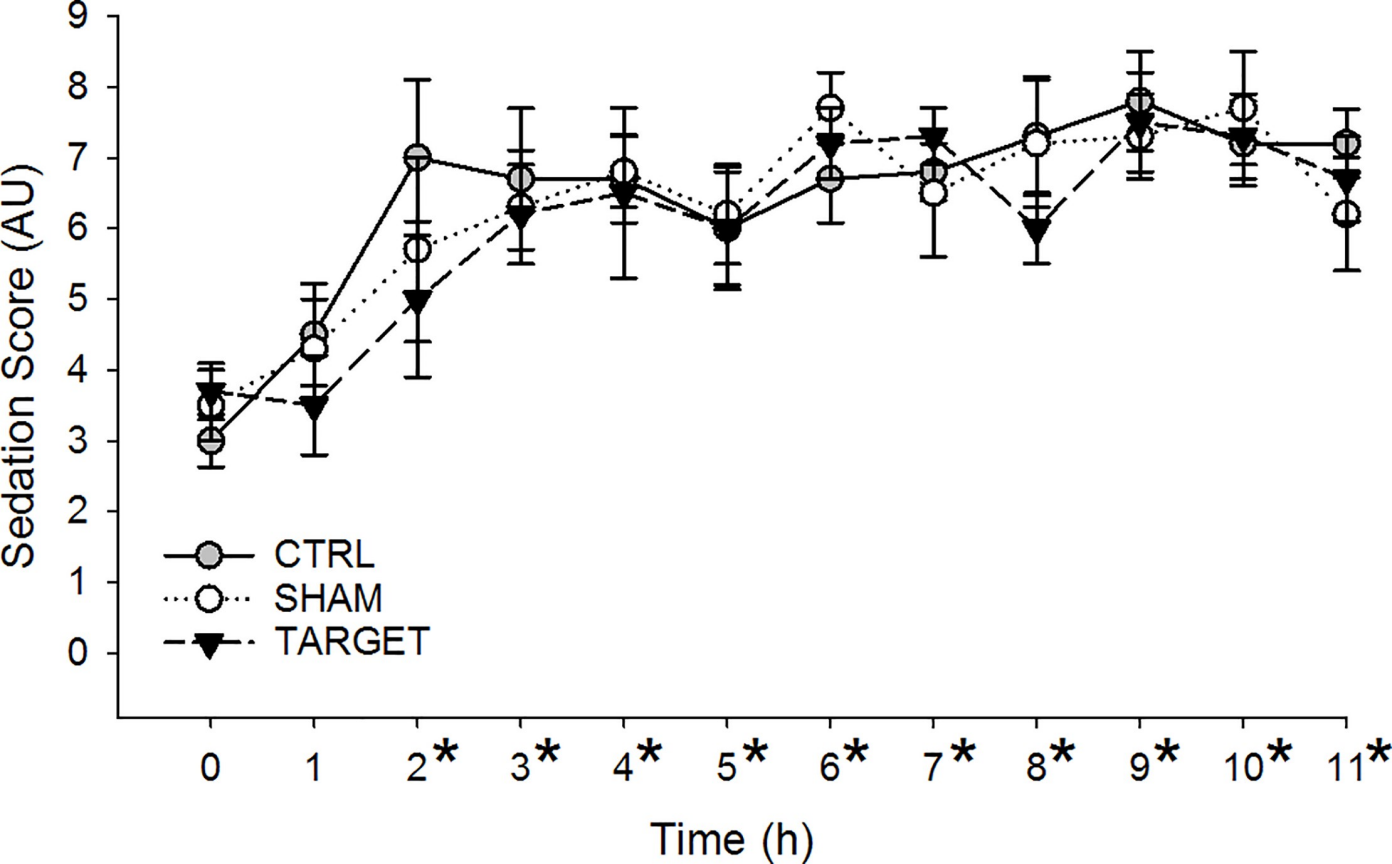
Sedation scores in healthy dogs given a loading dose of 1 mg/kg IV lidocaine followed by an infusion of 50 μg/kg/min. Dogs were either given no acupuncture (gray circles; CTRL), acupuncture at LU5 and BL55 (white circles; SHAM), or acupuncture at PC6 and ST36 (black triangles; TARGET). Scores ranged from 0 to 21 and are presented as mean ± SEM. No differences between treatment groups were present. *Significantly increased time point across treatment groups compared with Time 0.

A 22 gauge intravenous catheter (Zoetis Inc., Kalamazoo, MI) was placed either in the proximal cephalic or distal saphenous vein, avoiding acupuncture sites. Treatment randomization was determined by a computer-generated list (www.randomizer.org; generation date: 06/08/2018). Immediately after catheter placement, bilateral acupuncture was administered for 30 minutes either at: 1) PC6 and ST36 (TARGET; Fig 1), 2) Lung-5 and Bladder-55 (LU5 and BL55; SHAM; Fig 1), or 3) dogs had no acupoints stimulated (control, CTRL). Specifically, TARGET acupoint PC6 was located on the medial thoracic limb, 3 cm proximal to the transverse carpal crease, between the flexor carpi radialis and the superficial digital flexor muscles, and acupoint ST36 was accessed on the craniolateral pelvic limb 3 cm distal to the tibial plateau and 0.5 cm lateral to the tibial crest, within the belly of the cranial tibialis muscle. SHAM acupoint LU5 was located lateral to the biceps brachii tendon and medial to the brachialis muscle tendon in the cubital crease on the medial thoracic limb, and acupoint BL55 was accessed on the caudolateral pelvic limb between the biceps femoris and semitendinosus muscles caudal to the stifle. SHAM acupoints LU5 and BL55 were chosen as they have not been associated with or used for the treatment of nausea, vomiting, or GI dysmotility but are also limb points in similar locations to PC6 and ST36 [26, 32].

Needles were placed in a single motion with no additional manipulation and immediately secured with a loose strip of porous tape and light bandage covering sites, with care not to cause excessive compression of needles (PetFlex; Andover Medical Inc., Salisbury, MA); control dogs only had the bandage placed with no needles to ensure blinding of treatments. All techniques involving acupuncture were performed with 0.25 x 13mm, 3 gauge, surgical, stainless steel acupuncture needles (Hwa-To Singles, Suzhou Medical Appliance Factory, Suzhou, China) by the same two individuals trained in acupuncture (different investigators from the scorers). Scorers were blinded to all treatments and were not present for needle placement or removal. The same individuals placing needles removed bandages; no acupuncture needles were found displaced.

Dogs were returned to their housing units and immediately given a lidocaine (5mg/ml, Hospira Inc., Lake Forest, IL) loading dose (1mg/kg IV) within 2 minutes of needle placement, followed by infusion at 50 μg/kg/minute for 11 hours (Medfusion 3500 syringe pump; Smiths Medical Inc., Dublin, OH), similar to previously published protocols [13].

#### Radiographic interpretation, sedation and nausea scoring frequency

Nausea and sedation scoring was performed every hour thereafter (Times 1 through 11) while still in the housing unit. Nausea criteria involving an event (i.e. licking, swallowing, circling) and frequency of an event (i.e. number of vomiting or retching episodes) included observations throughout the prior 60 minutes. The interactive portion of sedation scoring at each time point was performed over the 5 minutes immediately prior to obtaining three-view radiographs, which were also taken hourly or until at least 90% of remaining BIPS were in the colon. Infusion pumps were never removed from the dogs and following each set of radiographs, dogs were quickly returned to their housing. At least 6 days elapsed between treatments.

A single blinded radiologist interpreted gastric emptying and GI transit times, which were determined at each time point by assessment of the location and number of BIPS in the stomach, small intestine, or large intestine [13, 25]. BIPS that passed distal to the ileum (cecum, colon, and rectum) were counted as in the large intestine. When BIPS location was uncertain as to whether it was in the stomach or small intestine due to superimposition, a conservative selection was made so that only BIPS located definitively in the small intestine were counted. Similarly, only BIPS located definitively in the large intestine were counted as such. Time for each event was recorded to the next hour because of the frequency of radiographic images.

### Statistical analyses

Statistical analyses were performed using SAS software version 9.4 (SAS Institute, Cary, NC). Since 6 dogs were repeatedly used in three treatments, the BIPS counts (number of BIPS outside stomach or in the colon/rectum), time for 25%, 50%, 75% and 90% of BIPS to empty from the stomach or reach the large intestine, and the sedation and nausea scores were all analyzed with a linear mixed-effect model using SAS PROC GLIMMIX. The absolute number of BIPS found outside of the stomach or in the large intestine were analyzed using a piecewise linear mixed-effect model to accommodate the beginning zero counts (number of BIPS in large intestine) and ending plateau (number of BIPS outside stomach) phases. The linear mixed-effect model can test differences between treatments at each time point and potential interactions between time and treatment. All data are presented as mean and standard error of the mean (SEM). All P values were 2-sided, and P < 0.05 was used to indicate statistical significance. If the overall P value was < 0.05, each time point was compared with Time 0, and the pair-wise P value was reported; if the overall time effect was not significant, no further comparisons were done.

## Results

### Nausea scoring

No statistically significant differences were found between treatments in nausea scores and there were no interactions between time and treatment (P = 0.1767 and P = 0.4124; Fig 2). However, an overall time effect was present (P < 0.0001) as mean nausea scores across treatment groups were significantly higher than Time 0 (0.2 ± 0.2) at Times 5 through 7 and Time 11 (2.4 ± 0.7, P = 0.0069; 4.4 ± 1.0, P < 0001; 2.6 ± 0.7, P = 0.0038; and 1.9 ± 0.7, P = 0.0342, respectively). All other time points were not significantly different than Time 0 (P > 0.05) when dropping the interaction of time and treatment.

### Sedation scoring

Similarly, no statistically significant differences were found between treatments in sedation scores and no significant interactions between time and treatment were present (P = 0.3741 and P = 0.8638; Fig 3). However, an overall time effect was present (P < 0.0001) as mean sedation scores across treatment groups were significantly higher than Time 0 (3.4 ± 0.2) from Times 2 through 11 (means range from the lowest value of 5.7 ± 0.7 at Time 2 to a maximum of 7.6 ± 0.4 at Time 9, all P < 0.0001). Sedation scores at Time 0 did not differ from Time 1 (4.3 ± 0.4, P = 0.1261) when dropping the interaction of time and treatment. Individual dogs scores in the TARGET and SHAM groups ranged from 1 to 11 and from 1 to 10 in the CTRL group; no dogs ever achieved profound sedation (score > 13) based on the scoring system used in this study.

### Gastric emptying and GI transit times

**Fig 4:**
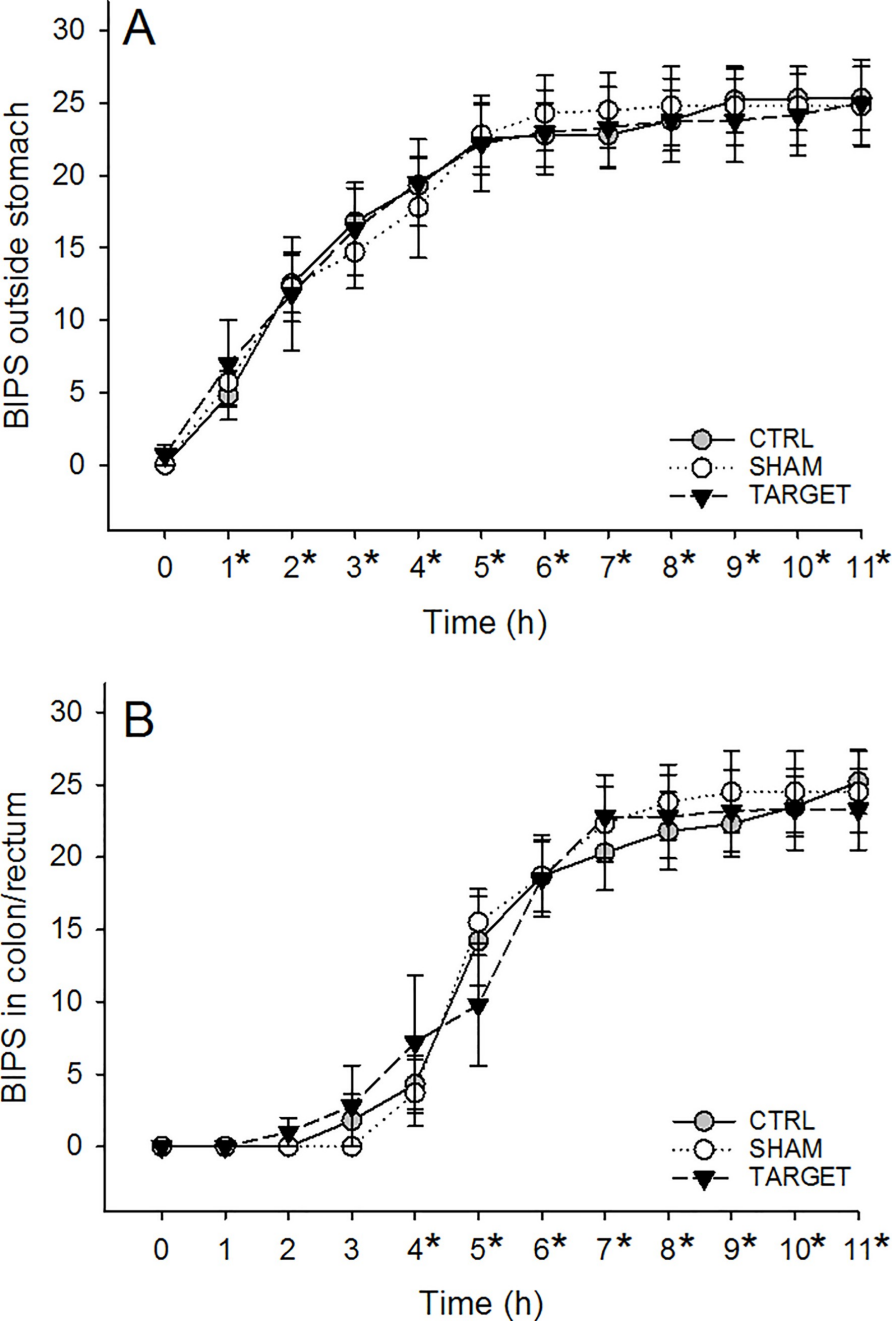
**The mean number** ± **SEM of BIPS located outside of the stomach (A) or within the colon/rectum (B) in healthy dogs fed 30 BIPS, prior to a loading dose of 1 mg/kg IV lidocaine followed by an infusion of 50 μg/kg/min.** The number of BIPS found outside the stomach and found within the colon/rectum did not differ between CTRL (gray circle, solid line), SHAM (white circle, dotted line), and TARGET (black triangles, dashed line) groups. *Significantly increased number across treatment groups compared with Time 0.

BIPS were easily identified and quantified throughout the study. Two different dogs in each treatment group vomited BIPS at various points during the study, resulting in less than 30 BIPS in their GI tract and the absolute number of BIPS remaining at the study endpoint (90% of ingested BIPS) differed in those dogs. No significant differences in the number of BIPS outside the stomach or in the colon/rectum were present between treatments (P = 0.9923 and P = 0.8958, respectively; Fig 4A and 4B). The average number of BIPS across treatment groups found outside the stomach was significantly increased from Time 0 (0.2 ± 0.9 BIPS) at Times 1 through 11 (averages range from 5.8 ± 5.1 BIPS at Time 1 to 25.1 ± 6.2 BIPS at Time 11, all P < 0.0001; Fig 4A). The average number of BIPS in the colon/rectum across treatment groups was significantly increased from Time 0 (0.0 ± 0.0 BIPS) only at Times 4 through 11 (Time 4: 5.1 ± 7.5 BIPS, P = 0.0025, all others range from 13.2 ± 8.0 BIPS at Time 5 to 24.4 ± 1.4 BIPS at Time 11, all P < 0.0001; Fig 4B).

In addition, no significant differences between treatments were found in the times for 25%, 50%, 75% and 90% gastric emptying of BIPS (all P ≥ 0.6329; Table 3) or in the times for 25%, 50%, 75% and 90% of BIPS to reach the large intestine (gastrointestinal transit times; all P ≥ 0.5240; Table 4).

**Table 3:** Time desired percentage emptied from stomach (gastric emptying time; hrs).

Treatment	25%	50%	75%	90%
CTRL	2.0±0.4	3.0±0.5	5.3±1.3	6.0±1.0
SHAM	2.2±0.6	3.5±0.6	4.5±0.3	5.8±0.6
TARGET	2.0±0.4	3.3±0.7	4.5±1.3	5.5±1.9

**Table 4:** Time desired percentage found in large intestine (gastrointestinal transit time; hrs).

Treatment	25%	50%	75%	90%
CTRL	4.7±0.4	5.8±0.7	7.5±1.3	8.0±1.1
SHAM	4.7±0.2	5.6±0.4	6.5±0.6	7.5±0.6
TARGET	5.0±0.5	5.7±0.6	7.0±1.5	7.5±1.7

No significant differences were found between treatment groups at any time point.

## Discussion

Contrary to the hypothesis, the results suggest that nausea and sedation associated with lidocaine infusions is not alleviated by acupuncture at PC6 and ST36 in healthy, conscious dogs. This explorational study was directed at investigating the use of acupuncture as a clinical tool in practice. However, before negating the efficacy of acupuncture in this regard, additional well-designed studies will need to be performed.

Quantifying nausea in dogs can be challenging due to dogs’ inability to express nauseous feelings and the subjective assessment of behavioral signs associated with nausea or other co-morbidities. For example, discomfort caused by alterations in GI motility may manifest as clinical signs similar to those seen with nausea, and dysmotility itself may contribute to nausea [21, 29]. To this end, although the scoring system used in this study had not been completely validated in dogs, both overt signs such as vomiting, as well as observance of numerous detailed behavioral signs as markers of nausea were used [adapted from: 20, 26, 29–33]. Basing clinical decisions solely on non-specific behaviors could result in treatments that include increasing the medications contributing to nausea itself (i.e. lidocaine or opioids), especially in post-operative patients. Although some assessments used to score nausea relied on observer interpretation and a degree of subjectivity, this study design using a single investigator to evaluate nausea and a more detailed, adapted scale from previously published scoring systems likely minimized these issues; the scale was effective in showing the onset, degree, and duration of nausea expected with lidocaine infusions in dogs.

In addition, the use of lidocaine infusions in dogs as a distinct model of nausea and vomiting has not been completely validated. However, nausea and vomiting associated with lidocaine infusions in dogs have been documented in previous investigations [8, 12, 13, 34] and occurred in the present study, similar to reports of opioid induced nausea and vomiting [26, 35]. As lidocaine is clinically used as an antiarrhythmic agent and is frequently administered peri-operatively in dogs, its associated side effects must also be considered; thus, treatments for these untoward effects should be investigated. The goal of our investigation was to investigate a clinical, non-invasive technique to reduce these significant side effects, which may limit the usefulness of commonly used lidocaine infusions in some situations.

Acupuncture is hypothesized to exert general effects through mechanisms including autonomic nervous system activation to restore homeostasis (nerve reflex theory), neurohumoral actions, and release of endorphins [5, 15, 16, 18, 21, 22, 36–38]. For example, acupuncture may reduce nausea through the above mechanisms within the area postrema and by reducing discomfort through normalizing gut motility [20, 26]. Although previous investigations suggest that acupuncture might mitigate changes in GI motility, which may contribute to nausea (13, 26), gastric emptying and GI transit times did not differ between groups in the present study. Nausea and vomiting are multifactorial events, dependent on the patient’s disease processes, anesthetics, the procedure performed, and co-morbidities [15, 16, 22, 39]. In addition, the vomition center contains groups of neurons in the medulla that includes the chemoreceptor trigger zone in the area postrema and the nucleus tractus solitarius, which can exert differing actions on vomiting when stimulated, for example, by pharmacologic agents [26, 31, 40]. We speculate that acupuncture at PC6 and ST36 may also have differing effects at these CNS areas and may not be altogether efficacious at the relevant physiologic pathways associated with lidocaine-induced nausea and vomiting. In addition, the physiologic effects of the acupuncture techniques used in the present study could have been either short acting compared with the duration of lidocaine infusion, or were relatively weak compared to the emetic stimulus associated with the clinically-used lidocaine infusion dose. In this regard, acupuncture may be beneficial when used as an adjunct to other antiemetic interventions. However, these hypotheses are yet to be tested.

In a previous study, nausea scores in dogs were significantly elevated by 4 hours during a similar lidocaine infusion and displayed a second elevation at Time 11 [13]. The dogs in this study did not show statistically significant signs of nausea/vomiting until 5 hours but did reveal a similar delayed elevation in scores at Time 11. Thus, it is possible that acupuncture at PC6 and ST36 mildly and transiently suppressed nausea and vomiting in the treatment group (for example, up to 4 hours); however, this effect is not substantial. In contrast, these data did not demonstrate differences in GI transit times between treatment groups, suggesting that lidocaine might mitigate the transient change in GI transit time associated with PC6 and ST36 acupuncture [25]. It is still possible that alterations in GI motility may play a role in nausea and vomiting; however, conclusions concerning data presented here should be made cautiously due to low statistical power (n = 6) although this sample size is commonly used in veterinary studies, has been used in prior foundational studies, and was adequate to find statistical significance in a similar investigation [13, 25].

Pharmacologically induced nausea likely differs mechanistically from other clinical manifestations of nausea associated with disruptions to homeostatic regulatory processes, such as that seen with pre-existing GI disease, which could explain differences in acupuncture efficacy. For example, acupuncture increases cerebrospinal levels of endogenous opioids (i.e. endorphins, enkephalins, endomorphins), which may modulate afferents to the vomition center in the brain [18, 19, 26, 33, 36, 41] and may have stronger effects on returning abnormal physiology to homeostasis in patients with active disease processes through actions on the autonomic nervous system and immune system [18, 21, 22, 41, 42]. Furthermore, serotonin, a signalling molecule active in the gastrointestinal tract, is altered after acupoint stimulation and may be involved in resolving gastrointestinal dysmotilies and nausea associated with pre-existing GI conditions [22, 41–43]. The above factors may explain, at least in part, the lack of efficacy on the drug induced model of nausea and vomiting used here. Furthermore, the results may differ in future studies addressing the effects that acupuncture and lidocaine infusions may have on GI motility, sedation, and nausea in post-operative dogs with homeostatic perturbations or in conscious dogs with pre-existing abnormal GI function or motility.

Sedation is not only associated with lidocaine infusions in dogs [12, 13], but also with acupuncture itself [36, 38, 44, 45]. Glutamate, due to modifications in receptor expression, and serotonin, through increased action on spinal receptors and decreased binding to cortical receptors, are suspected to contribute to the analgesic and sedative properties of acupuncture [18, 37, 41]. In the present study, there were no significant differences in sedation scores between groups and mean sedation scores in most groups were considered moderate as described by a validated scale [31]. However, all groups demonstrated a significant increase in sedation beginning two hours from the start of lidocaine infusion, suggesting that sedation may be due to the lidocaine infusions, which may have masked any subtle differences between acupuncture and control groups. However, all the sedation is difficult to exclusively link to lidocaine as the sedation score 1 hour after the bolus (Time 1) was not statistically different from Time 0 as would be expected if the sedation was solely associated with lidocaine. Although we speculate that accumulating plasma lidocaine levels increased sufficiently following the bolus and start of the infusion to cause significant sedation by Time 2, it cannot be ruled out that although all dogs were acclimatized and handled by the same individuals throughout experimentation, and mean baseline (Time 0) scores were at the low end of the moderate sedation scores (3.4 ± 0.2), their excitement level may have been higher at the beginning of each study and they became increasingly cooperative during the experiment.

Various acupoints could have been chosen to target viscera (i.e. CV12, BL20). Because CV12 is on ventral midline (between the umbilicus and xyphoid process) with only one acupoint (not bilateral) and BL20 is bilateral on the back (in epaxial muscles near the last rib), blinding by covering with light bandages would be difficult. Additionally, limb acupoints are easily accessible and acupuncture is easily achieved there in most clinical scenarios. However, the lack of treatment efficacy may have been due, at least in part, to our choice of acupoints.

A potential limitation of this study may be the lack of a “positive” control group, potentially administrating an anti-emetic agent used for many types of canine nausea and vomiting such as maropitant citrate [46]. Addition of this experimental group may have helped assess the adequacy of our measurement techniques used to quantify nausea and vomiting since maropitant would be expected to reduce vomiting. However, the use of pharmacologic agents specifically directed at improving nausea and vomiting associated with lidocaine infusions has not been previously confirmed.

Acupuncture efficacy is often improved if applied as an early treatment modality, thus, acupuncture was performed immediately prior to lidocaine infusion in this study [15, 19, 23, 45]. The acupuncture points and duration in this study were chosen based on previous investigations [13, 15, 17, 21, 25, 26], but may have been inadequate for the degree of nausea and vomiting induced in this experimental model, only resulting in partial or transient effects as discussed above. Furthermore, the type of acupuncture stimuli applied may have affected treatment efficacy. For example, electrical acustimulation is more effective than traditional needle acupuncture and may have been more effective in our study [15, 16, 20, 24, 37, 45].

## Conclusions

In conclusion, stimulation of PC6 and ST36 acupoints alone are inadequate to significantly alleviate nausea and vomiting in healthy dogs administered lidocaine infusions. However, these results may differ when these acupoints are used in patients with underlying GI pathology or if more efficacious acupuncture techniques are employed.
